# A robust multimodal detection system: physical exercise monitoring in long-term care environments

**DOI:** 10.3389/fbioe.2024.1398291

**Published:** 2024-08-08

**Authors:** Naif Al Mudawi, Mouazma Batool, Abdulwahab Alazeb, Yahay Alqahtani, Nouf Abdullah Almujally, Asaad Algarni, Ahmad Jalal, Hui Liu

**Affiliations:** ^1^ Department of Computer Science, College of Computer Science and Information System, Najran University, Najran, Saudi Arabia; ^2^ Department of Computer Science, Air University, Islamabad, Pakistan; ^3^ Department of Computer Science, King Khalid University, Abha, Saudi Arabia; ^4^ Department of Information Technology, College of Computer and Information Sciences, Princess Nourah Bint Abdulrahman University, Riyadh, Saudi Arabia; ^5^ Department of Computer Science, Faculty of Computing and Information Technology, Northern Border University, Rafha, Saudi Arabia; ^6^ Cognitive Systems Lab, University of Bremen, Bremen, Germany

**Keywords:** accelerometer, convolutional neural network, exercise detection, gated recurrent units, deep learning, multimodal human activity recognition, smart inertial measurement unit, GPS sensor

## Abstract

**Introduction:**

Falls are a major cause of accidents that can lead to serious injuries, especially among geriatric populations worldwide. Ensuring constant supervision in hospitals or smart environments while maintaining comfort and privacy is practically impossible. Therefore, fall detection has become a significant area of research, particularly with the use of multimodal sensors. The lack of efficient techniques for automatic fall detection hampers the creation of effective preventative tools capable of identifying falls during physical exercise in long-term care environments. The primary goal of this article is to examine the benefits of using multimodal sensors to enhance the precision of fall detection systems.

**Methods:**

The proposed paper combines time–frequency features of inertial sensors with skeleton-based modeling of depth sensors to extract features. These multimodal sensors are then integrated using a fusion technique. Optimization and a modified K-Ary classifier are subsequently applied to the resultant fused data.

**Results:**

The suggested model achieved an accuracy of 97.97% on the UP-Fall Detection dataset and 97.89% on the UR-Fall Detection dataset.

**Discussion:**

This indicates that the proposed model outperforms state-of-the-art classification results. Additionally, the proposed model can be utilized as an IoT-based solution, effectively promoting the development of tools to prevent fall-related injuries.

## 1 Introduction

Falling is one of the most significant global public health issues (Maritta et al., 2021). With an estimated 646,000 fatal falls occurring worldwide each year, falls are the second leading cause of unintentional injury deaths (Singh et al., 2021). This is particularly detrimental to the elderly, who progressively lose their ability to control their movements smoothly with advancing age (Yang et al., 2022). The threat to public health is further exacerbated by the growing elderly population (Wang et al., 2024). Recently, the World Health Organization (WHO) reported that deaths from falls most common among individuals over the age of 65 years, with a significant prevalence also noted in adults over the age of 60 years (Zhang et al., 2023).

In the modern era, fall detection has become a key and vital research area within the investigation of biosurveillance systems (Zhao et al., 2024). The duration that individuals remain on the floor after falling plays a crucial role in determining the severity of the fall (Wan et al., 2020). Early identification of falls enables caregivers to provide prompt assistance, thereby reducing the negative effects of falls (Dua et al., 2021). A reliable fall detection biosurveillance system can significantly relieve the strain on caregivers by monitoring falls and sending out timely notifications (Mahmood et al., 2020; Zhou et al., 2020; An et al., 2022; Velliangiri et al., 2020).

In the modern era of artificial intelligence, fall detection holds significant promise; yet, numerous challenges remain (Chen et al., 2020). A fall is typically defined as an incident that causes a person to unintentionally come to rest on the ground, a floor, or another lower level while engaged in physical activity (Gochoo et al., 2021). However, fall detection is a comparably challenging task due to the prevalence of similar behaviors in daily life activities (Wang et al., 2023). For instance, lying down is a common action that can complicate fall detection (Khalid et al., 2021). Additionally, falls are random, unplanned, and harmful events, making it difficult to collect genuine data during daily physical activities (Cai et al., 2023).

Numerous scholars have studied the use of multimodal sensors to evaluate and observe fall detection, particularly in elderly populations. Ghadi et al. (2022) presented a wavelet pattern recognition and multi-feature extraction methodology to extract multisensory features. These features were then optimized and classified using fuzzy logic-based optimization and a hidden Markov model (HMM) to detect falls and daily life activities, achieving an accuracy of 87.5% on the UP-Fall Detection dataset. Villaseñor et al. (2019) gathered a fall detection dataset using wearable, ambient, and camera sensors. Their technique used correlation-based feature selection and applied three machine learning and neural network algorithms, including k-nearest neighbors, support vector machines (SVMs), random forests, and multi-layer perceptrons. The proposed technique achieved the highest accuracy of 95.1% using the random forest classifier on the UP-Fall Detection dataset. Marvi et al. (2021) introduced a noise-tolerant fall detection system designed to remain effective even in the presence of missing data. They utilized a deep learning framework, specifically a recurrent neural network and bidirectional long short-term memory (LSTM), on wearable sensor data. This technique achieved an impressive accuracy of 97.41% on the UP-Fall Detection dataset. Kraft et al. (2020) presented a harmonized fall detection technique tested on the MUMA, SimFall, and UP-Fall Detection datasets. Their harmonization strategy involved cropping a 200-timestamp window from the MUMA dataset and a 250-timestamp window from the central point of the SimFall dataset time series. Additionally, a peak detection algorithm was applied to the UP-Fall Detection dataset. This deep learning algorithm achieved a top-notch accuracy of approximately 93.3% on these benchmark datasets.

Gasparrini et al. (2016) developed a TST Fall Detection dataset using camera and wearable sensors. A data fusion approach was applied to this dataset, and the variation in skeleton joint positions yielded an accuracy of approximately 90%. Yao et al. (2019) used traditional features, which were later classified with SVM. By adjusting the threshold through extensive experiments, this feature-based method combined with machine learning technology achieved an impressive accuracy of 93.56% on the TST Fall Detection dataset. Seredin et al. (2019) used skeleton-based features to encode information from neighboring frames. These individual decisions from neighboring frames were combined using the cumulative sum method, and an SVM classifier was then applied to the resultant data. This model achieved an accuracy of 95.8% on the TST Fall Detection dataset. The system was validated using a leave-one-person-out cross-validation scheme. Mendez et al. (2019) identified human orientation features selected by a genetic algorithm to determine the posture of a skeleton when it is about to fall or is close to the floor. Utilizing SVM, along with velocity and acceleration, they obtained an accuracy of 90% on the TST Fall Detection dataset.

To improve feature capture, two-stream convolutional neural networks (CNNs) have been applied to fall detection. Fei et al. (2023) used optical flow input into VGG-16 for temporal feature capture and key nodes of the human skeleton input into ST-GCN for spatial feature capture. The process then involves feature fusion and binary classification. Significantly, this detection method remains unaffected by lighting variations and shows enhanced robustness. There is growing research interest in integrating the CNN and LSTM networks. Apicella et al. (2021) used PoseNet for pose estimation and a pre-trained CNN to generate additional poses in the cases where skeletal key points are absent or pose estimation encounters difficulties. The subsequent classification task is managed by LSTM. This modular architecture facilitates the easier implementation of enhancements and adjustments. Inturi et al. (2023) explored the spatial correlation of acquired skeletal key points using the CNN while also maintaining long-term dependencies through LSTM networks. Their study underscores the superior accuracy of AlphaPose in detecting key points compared to OpenPose.

In this paper, the multimodal sensor data have been first pre-processed separately. Then, wave length features and autoregression have been used to extract features of inertial sensor data. Additionally, Markov random field (MRF) and ridge regression have been used for depth and RGB sensor data. The principal component analysis (PCA) has been used to fuse the data of multimodal sensors. Finally, a convolutional neural network–gated recurrent unit (CNN-GRU) has been used for the prediction of fall detection. It could also be used for the classification of different daily life activities.

The primary contributions of our paper are as follows:

• The selection of features from various domains aims to optimize the extraction of relevant raw data information for classification tasks. This process simultaneously minimizes variations within classes and enhances distinctions between classes.
• To address the intricate patterns of movement dysfunction present in the fall detection dataset and enhance the identification rate of two benchmark datasets, an implemented approach involved the utilization of a modified K-Ary entropy classifier (KEC).
• Furthermore, a comprehensive comparative analysis was conducted on two publicly accessible and standardized datasets for fall detection, namely, UP-Fall Detection and TST Fall Detection datasets. The experimental results of the proposed model exhibited a high recognition accuracy rate in terms of perception efficacy compared to other cutting-edge methodologies.

The rest of the paper is organized as follows: Section 2 provides a description of the solution framework, encompassing pre-processing, feature extraction, feature fusion, and classification; Section 3 presents the experimental results, including a comparison to similar cutting-edge fall detection systems; and finally, Section 4 presents the conclusion and outlines potential future study areas.

## 2 Materials and methods

### 2.1 System methodology

**FIGURE 1:**
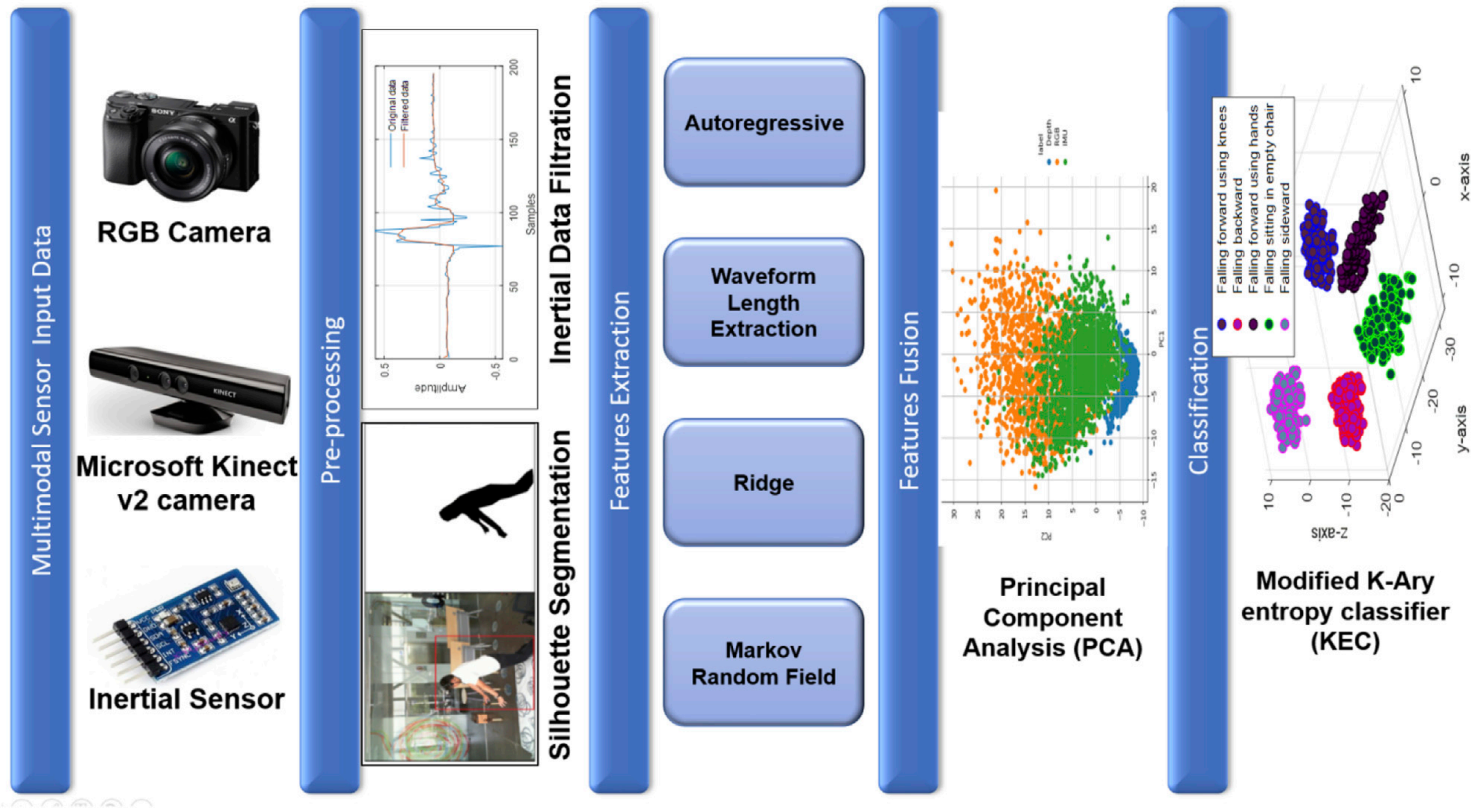
Comprehensive architecture of the proposed fall detection system.

Figure 1 depicts the proposed fall detection model for the fall activity detection of multimodal sensor data. The architecture of the proposed fall detection system begins with data acquisition, where RGB, depth, and inertial data were collected from the UP-Fall Detection and TST Fall Detection datasets. The UP-Fall Detection dataset includes both RGB video data and inertial sensor data capturing various fall activities. In contrast, the TST Fall Detection dataset contains accelerometer data and depth sensor data, specifically obtained from scenarios designed for fall activity detection within the TST framework. Both datasets provide comprehensive multimodal data, essential for developing and testing robust fall detection algorithms. Next, during the pre-processing phase, the Dynamic Data Reconciliation (DDR) filter was applied to clean the inertial data. Additionally, silhouette segmentation was performed on the RGB and depth data to extract silhouettes from the images for further processing. In the feature extraction process, autoregressive and waveform length methods were used to extract features from the inertial data. For the RGB and depth data, features were extracted using ridge regression and MRF techniques. Finally, the data extracted from various modalities were combined to bolster the effectiveness of the modified K-Ary entropy classifier through PCA.

### 2.2 Pre-processing

Pre-processing is an essential step of activity recognition and classification in the human fall detection procedure (Lu et al., 2022). It helps denoise the inertial data and extract the silhouette of RGB and depth images, thus aiding in ensuring optimal performance and accuracy (Khan et al., 2024). The pre-processing strategy of inertial, RGB, and depth sensors is briefly described below.

#### 2.2.1 Inertial sensor data

**FIGURE 2:**
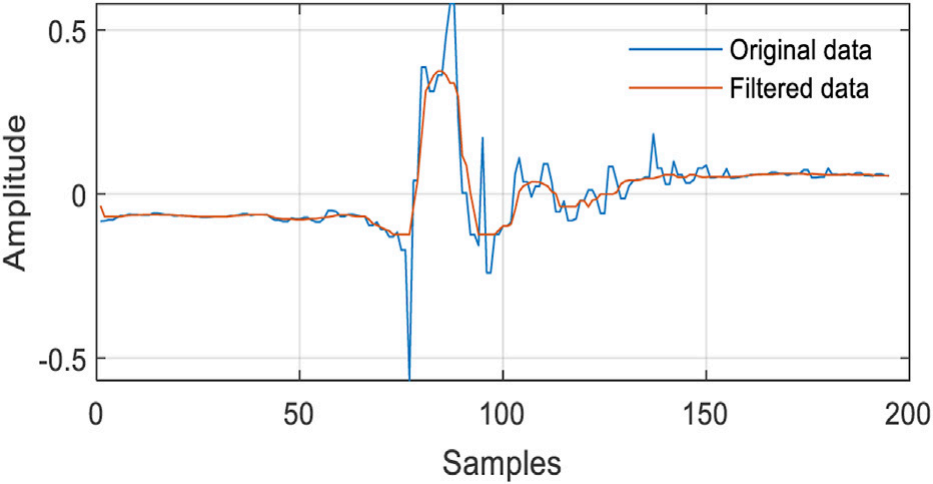
Visual representation of the ridge data edge detection and distance map over the UP-Fall Detection dataset.

Due to excessive sensitivity of inertial wearable sensors, the data contain random noise, which adversely affects the feature extraction process (Jalal et al., 2019a; Jalal et al., 2019b; Jalal et al., 2019c; Jalal et al., 2019d). Therefore, a filtration process is highly needed to mitigate the randomness of inertial sensor data (Liu et al., 2021). In this step, the DDR filter is applied to remove unnecessary randomness and noise inherited in the inertial data (Shi et al., 2022). The DDR filter works on the reconciliation principle by integrating the sample information of signals with respect to time *t* and window *w*. However, the main limitation of the DDR filter is to have the input data long enough to obtain the appropriate results. Therefore, 1-min-long inertial data samples have yielded standard results, as shown in Figure 2.

#### 2.2.2 RGB sensor data

**FIGURE 3:**
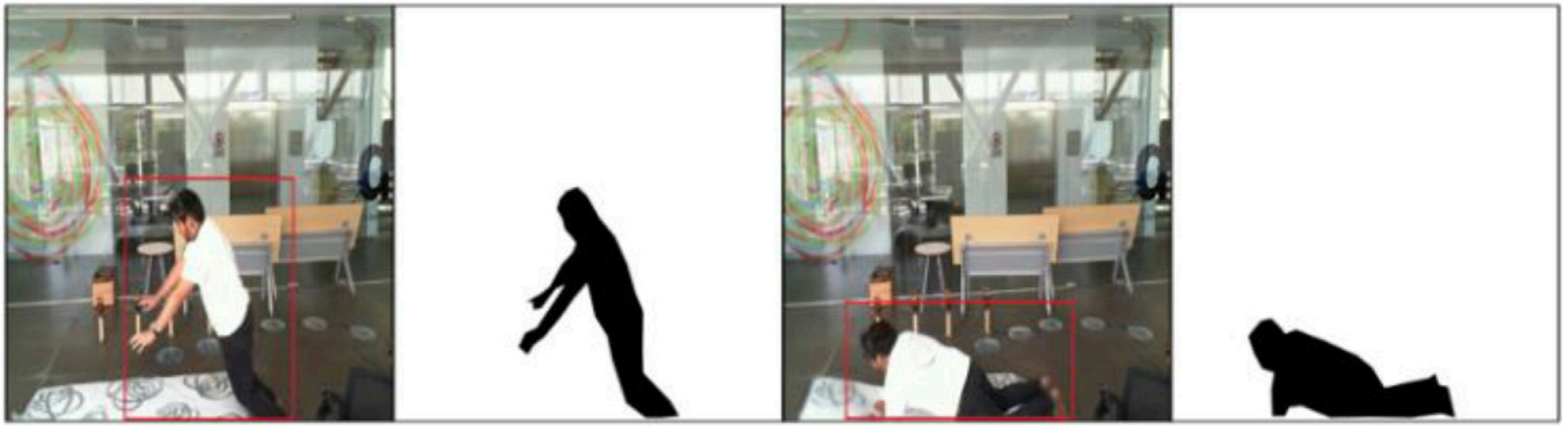
Graphical depiction of waveform length extraction on the UP-Fall Detection dataset.

In this process, the noise of RGB images has been removed using the median filter. Then, silhouette segmentation on RGB images has been performed by locating the components on the images using the four-connected pixel analysis algorithm (Cui et al., 2022). Then, bounding has been identified by applying a certain threshold over the height and width of the human body (Fu et al., 2023). Then, threshold segmentation has been applied to obtain the accurate results of RGB images (Liu et al., 2022). Finally, the silhouette from the depth image has been extracted using saliency map-based silhouette segmentation. The results are shown in Figure 3.

#### 2.2.3 Depth sensor data

**FIGURE 4:**
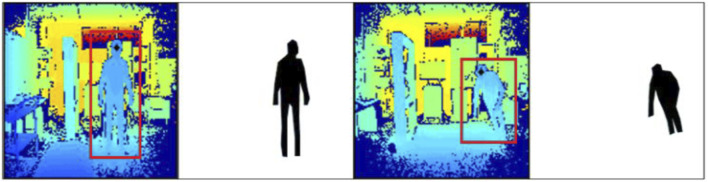
Graphical depiction of waveform length extraction on the TST Fall Detection dataset.

The silhouette of depth images has been extracted by first passing the depth images through the median filter (Batool et al., 2019). Then, the depth image has been converted to a binary image using Otsu thresholding (Sun et al., 2024). Then, the morphological operation of dilation and erosion has been applied to detect the contour of the images. The canny edge detection process has been applied to detect the edges of the human body. Then, extra objects have been removed from the depth image. Finally, the silhouette from the depth image has been extracted using saliency map-based silhouette segmentation. The results are shown in Figure 4.

### 2.3 Feature extraction

The feature extraction process is a vital part of the human activity classification process; it could either be the recognition of daily life activities, smart home activities, or elderly fall detection. The feature extraction methodologies vary with the type of sensors, such as time–frequency features and wavelet features, which have been mostly used for inertial sensor data. Moreover, HOG features, orientation features, and spatiotemporal features have been mostly used for RGB and depth sensor data (Gdairi et al., 2022). In this paper, autoregressive waveform length features have been used for inertial sensor feature extraction. Moreover, MRF and ridge regression have been used for RGB and depth feature extraction. The feature extraction process is briefly described below.

#### 2.3.1 Autoregressive features

**FIGURE 5:**
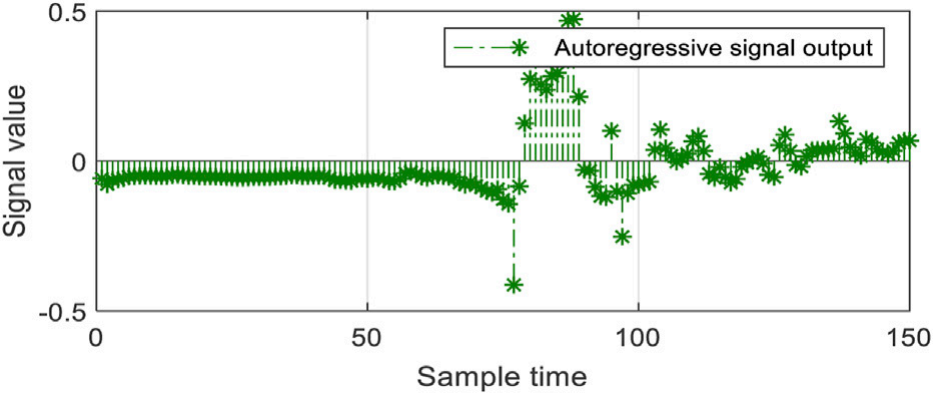
Calculated autoregressive features of the inertial data sensors over the UP-Fall Detection dataset.

The samples of each inertial sensor data frame have been represented by autoregression as a linear mixture of previous samples (Zhang et al., 2019) and white noise, as shown in Figure 5. The AR has been represented as

$$A_{r}\left(y\right)=\sum_{j=1}^{N}c_{j}y_{j-1}+e_{r},$$

where $A_{r}$ determines the coefficients of the $y_{j-1}$ sample, $e_{r}$ depicts the error rate, and N represents the order of the AR model. Here, a 13th-order polynomial has been used to obtain the resultant autoregressive coefficients.

#### 2.3.2 Waveform length extraction

The waveform length assesses the overall variance of the inertial signal by calculating the absolute difference of adjacent frames of the data in the signal (Jalal et al., 2020). The resultant scalar values are helpful in calculating the difference between high and low intensity of the inertial sensors data:

$$W_{L}\left(y\right)=\sum_{k=0}^{k-1}\left|y_{k+1}-y_{k}\right|,$$

where $y_{k+1}$ determines the anticipated value of the predicted signal and $y_{k}$ is the current value of the sample signal.

#### 2.3.3 Ridge

The ridge feature is one of the key feature extraction processes of RGB and depth silhouettes. The ridge involves two key components of binary edge extraction and distance map of the visionary data (Badar et al., 2020; Jalal et al., 2018). First, binary edge data have been detected by calculating the local statistical characteristics of each neighboring data of the depth silhouette. Then, a window has been applied to extract binary edge data surrounding these objects. Consequently, a well-defined body structure and robust edge connectivity have been obtained as

$${\;E}_{b}\left(ft\right)=\left\{{fp}_{c}\in ft|\exists {fp}_{i},\left|{fp}_{i}-{fp}_{c}\right|>\delta_{e}\right\},{fp}_{i}\epsilon \left\{{fp}_{c-1},{fp}_{c+1},{fp}_{c-w},{fp}_{c+w}\right\},$$

where ${fp}_{c}$ depicts the center of the depth pixel compared to its corresponding ${fp}_{i}$ adjacent depth pixels.

**FIGURE 6:**
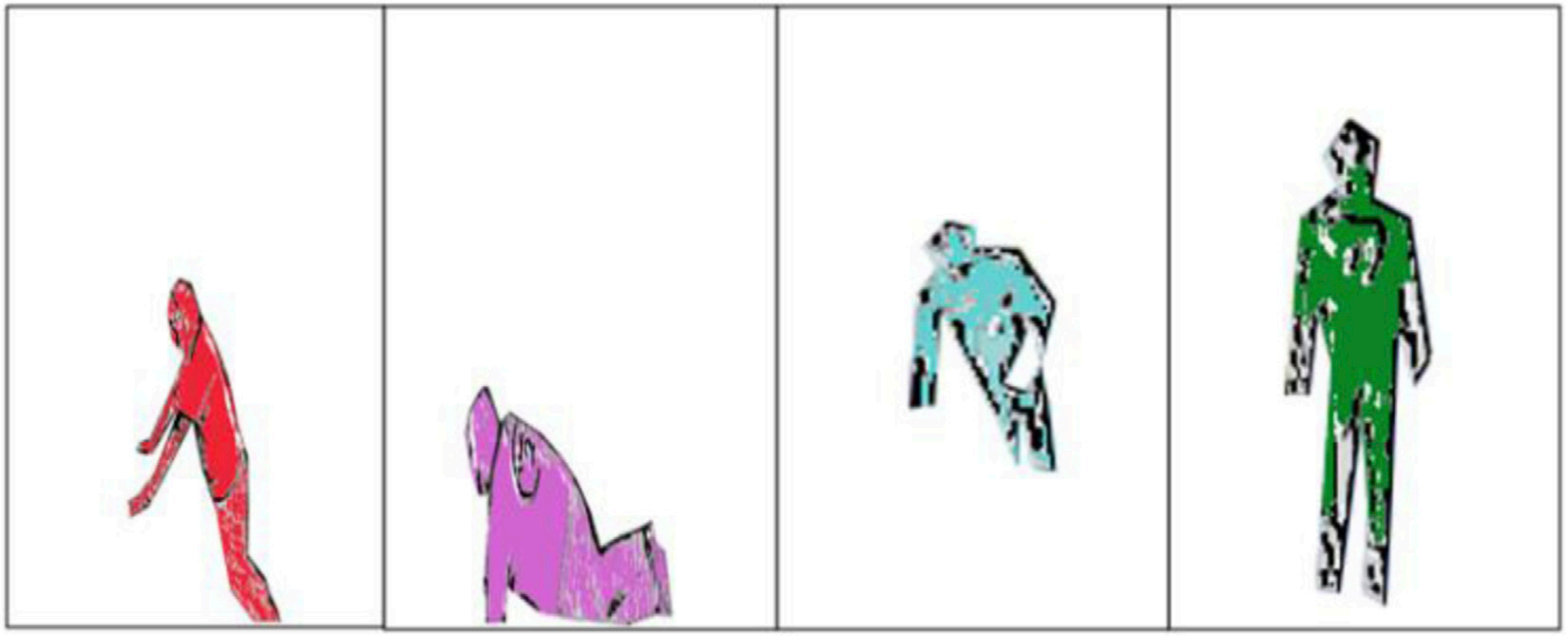
Visual representation of the ridge data edge detection and distance map over the UP-Fall Detection dataset.

Second, the distance map of the ridge data has been determined to obtain the local maxima of corresponding edges as a chain of pixels. The resultant ridge data surrounded by binary edges emulate the human skeleton.

$$D_{r}\left(ft\right)=\left\{{fp}_{c}\in \text{ft}\left|\frac{\sum_{k=1}^{n}F_{M}\left({fp}_{i}\right)}{\left(n\right)\left(F_{M}\left({fp}_{c}\right)\right)}\right.<\delta_{e}\right\},$$

where $F_{M}$ depicts the distance map of center pixels to the corresponding surrounding pixels. The ridge data results, as shown in Figure 6, effectively remove the noisy edge data and detect the position of the skeleton.

#### 2.3.4 Markov random field

**FIGURE 7:**
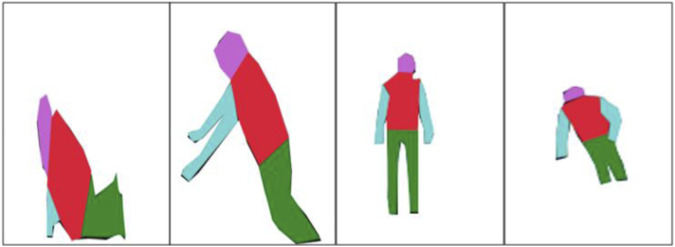
Visual representation of the ridge data edge detection and distance map over the UP-Fall Detection dataset.

The MRF merged the same-color regions in the RGB silhouette to maintain consistency of the segmented region (Chen et al., 2021). The MRF works by calculating the probability distribution of similar interacting features as

$$R_{c}=\left(S_{n},N_{b},D_{r}\right),$$

where $R_{c}$ depicts the relational vector of corresponding nodes, $S_{n}$ represents set of similar nodes, $N_{b}$ is the used neighboring nodes, and $D_{r}$ depicts the third degree of relationship across the adjacent nodes. The results of the MRF are shown in Figure 7.

### 2.4 Principal component analysis

**FIGURE 8:**
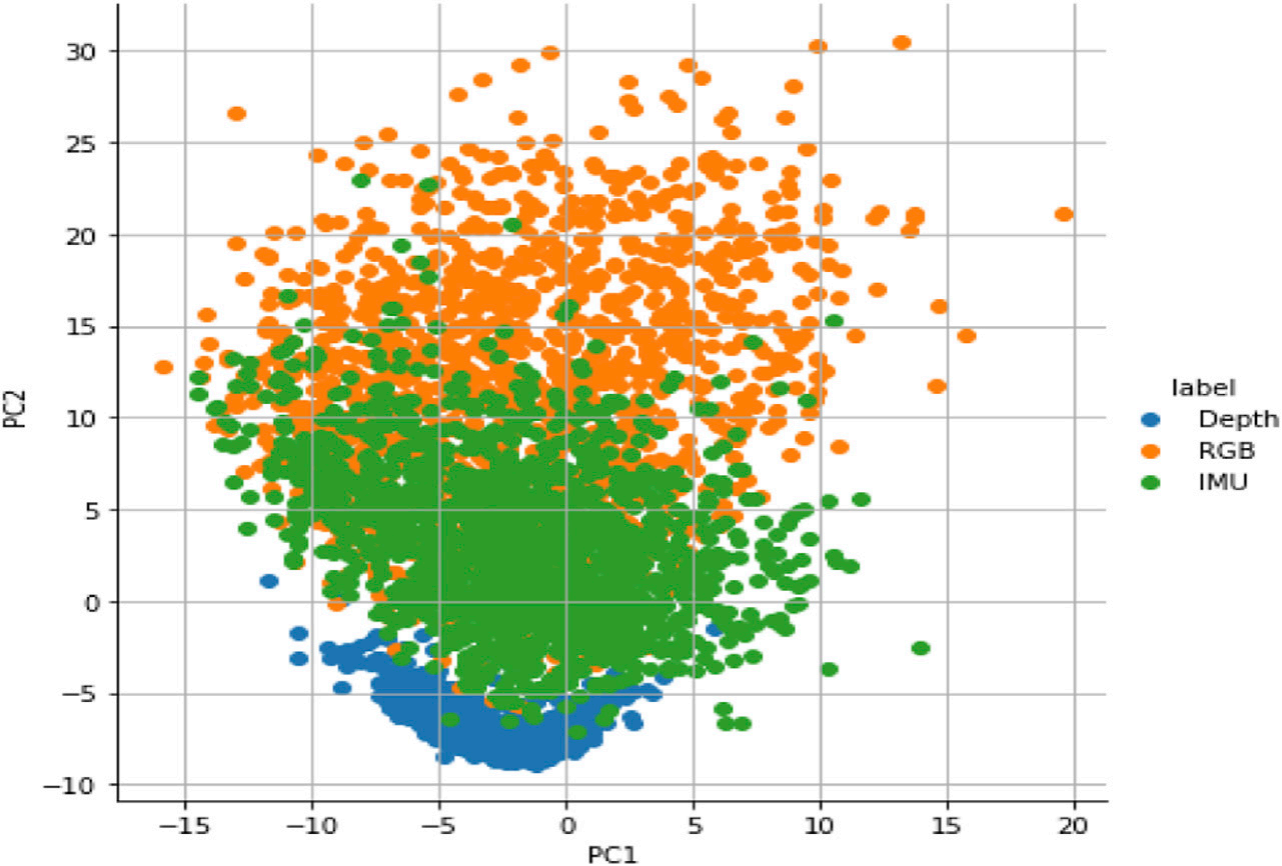
Principal component analysis (PCA) fusion results of multimodal sensor data.

At this stage, sensor data fusion is probably required to combine the data of multimodal sensors including RGB, depth, and inertial sensors into ubiquitous representation (Khatun et al., 2018; Kraft et al., 2020; Liu et al., 2022; Liu et al., 2021; Lu et al., 2022; Mahmood et al., 2020; Maritta et al., 2021; Marvi et al., 2021) (Prati et al., 2019; Jalal et al., 2019a; Jalal et al., 2019b; Jalal et al., 2019c; Jalal et al., 2019d; Khatun et al., 2018; Batool and Javeed, 2022a; Batool and Javeed, 2022b). PCA is a standard technique that systematically combines the data of different sensor modalities into ubiquitous format. PCA utilizes orthogonal information about multi-sensor data to preserve the least square average of the sensor data, hence eliminating the rest of the sensor data, as shown in Figure 8. This process has been conducted in three steps. First, the mean of the data has been calculated and then subtracted from each attribute $x_{i}$ of the data to obtain the center of the data:

$$M\left(x\right)=\left(x_{1},x_{2},,..........,x_{n}\right)-mean.$$

Second, the details about data variance and covariance have been obtained by computing the transpose of the covariance matrix. Finally, the eigenvalue has been selected with its corresponding eigenvector to obtain the effective result of multimodal sensor data.

$$\sum A_{mp}=\gamma A_{mp}.$$

### 2.5 Modified K-Ary entropy classifier

The modified KEC is our modified K-Ary hashing classifier (Wu et al., 2018) and has been shown to achieve a cutting-edge accuracy by Batool et al. (2023). In this paper, we have used the KEC for depicting the performance of multimodal sensor data over benchmark fall detection datasets. The resultant fused data of PCA have been given as input to the KEC. The KEC has been implemented using a one-level entropy-based hashing mechanism that divided the data into uniformly distributed subtree patterns. Moreover, Euclidean and hamming distances have been calculated to obtain the final classification results. The KEC algorithm first divided the entire vector array V into an approximately half-array as $2^{n}$ .

$$V\left(y\right)=\lceil \frac{y-\min\;\left(V\right)}{\max\left(v\right)-\min\;\left(v\right)}\times 2^{n}\rceil -1.$$

Next, the center of the vector array acted as a parent node, and the rest of the nodes acted as child nodes. The overall data of the vector array were then normalized into a single integer using the BitBooster technique. The distance between the two nearest nodes was then determined using the Euclidean distance, and the number of high bits in an integer was determined using the hamming weight. Euclidean and hamming distances together produced findings that are roughly accurate.

$$D_{vn}=\sqrt{H_{w}\left\{E_{d}\left(v_{1},v_{2},\ldots \ldots ,v_{n}\right)\right\}}.$$

**FIGURE 9:**
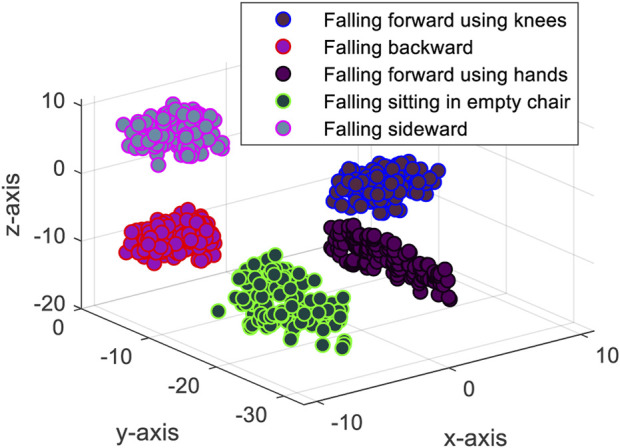
Classification accuracy of the modified K-Ary entropy classifier over the UP-Fall Detection Dataset.

The subtree patterns were finally categorized by calculating the inter-cluster entropy function, which distinguished between two or more subtree patterns, and within-cluster pattern entropy, which calculated the entropy of nodes within the subtree pattern. The final output of the classifier is shown in Figure 9.

## 3 Experimental results and dataset description

The proposed system has been built on an Intel Core CPU i5, 64-bit operating system with 8 GB RAM. The main coding has been carried out in Python using different signal and image processing techniques. The leave-one-out cross-validation scheme has been applied to measure the accuracy, recall, and F1 score. Moreover, UP-Fall Detection and TST Fall Detection benchmark datasets have been used for the fall detection mechanism of the biosurveillance system. Detailed information about these datasets is provided below.

### 3.1 UP-Fall Detection dataset

**FIGURE 10:**
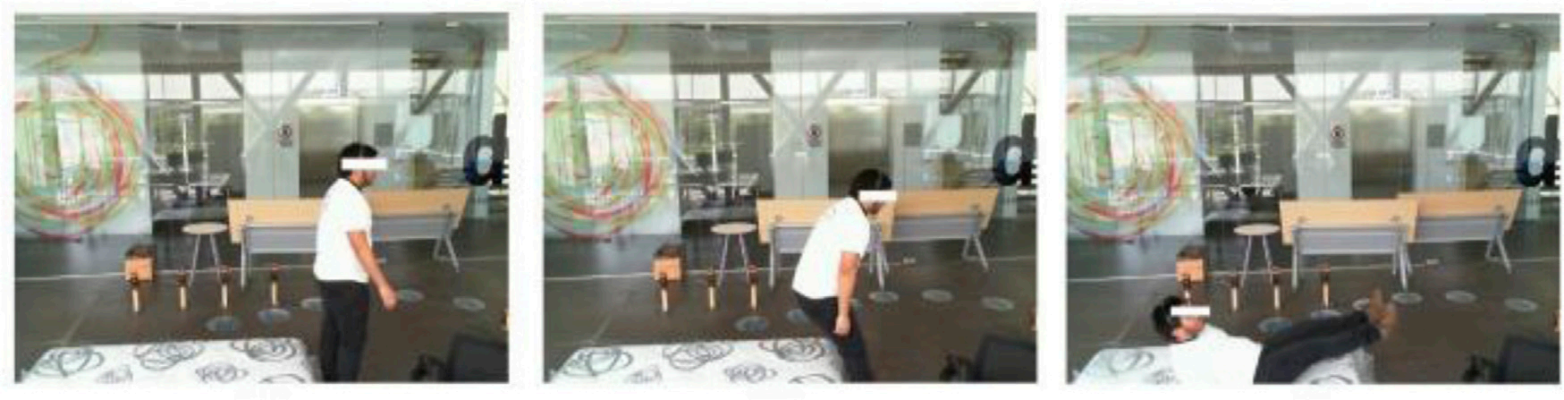
Sample images from the UP-Fall Detection dataset capturing instances of individuals falling backward.

The UP-Fall Detection dataset (Villaseñor et al., 2019) was collected using multimodal sensors including RGB and inertial sensors. During the data collection phase, a cohort of 17 young, healthy individuals devoid of impairments, comprising 9 males and 8 females, with ages ranging from 18 to 24 years, were enlisted. These subjects boasted a mean height of 1.66 m and a mean weight of 66.8 kg. They were tasked with executing 11 distinct activities, encompassing 6 routine human movements (walking, standing, picking up an object, sitting, jumping, and lying down) and 5 types of human falls (falling forward using hands, falling forward using knees, falling backward, falling while seated in an empty chair, and falling sideways). In this paper, we only used human fall activities, as shown in Figure 10. To mitigate potential injuries, a mattress was placed in the fall zone for all activities involving falls. The data collection process adopted a multimodal approach, incorporating wearable sensors, ambient sensors, and vision devices. However, in the proposed fall detection system, we only incorporated wearable sensors and vision RGB data. The data collection occurred on the third floor of the Faculty of Engineering at Universidad Panamericana, Mexico City, Mexico. All measurement devices and equipment were interconnected to a set of local computers, which served as a centralized hub for data consolidation. The collected data were then stored in hard drives for subsequent analysis. Notably, the dataset included a total of 384 RGB images, extracted from this meticulous data collection effort and used in the proposed fall detection system.

### 3.2 TST Fall Detection dataset

**FIGURE 11:**
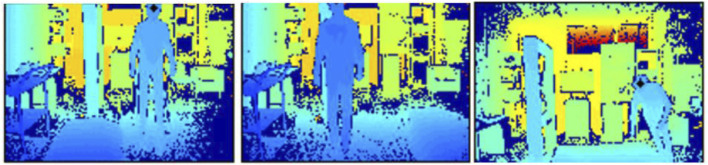
Sample images from the TST Fall Detection dataset capturing instances of individuals falling forward.

The dataset (Cippitelli et al., 2016) comprises 11 young, healthy volunteers aged between 22 and 39 years, with heights ranging from 1.62 m to 1.97 m. The dataset includes two main groups of actions: activities of daily living (ADLs) and fall-related activities. ADLs involve actions like sitting (where the actor sits on a chair), grasping (walking and picking up an object from the floor), walking (back-and-forth movement), and lying down. Fall activities include falling forward, backward, to the side, and ending up either lying down or sitting. In the proposed fall detection system, only fall activities are incorporated in Figure 11. The complete database contains 264 different actions, totaling 46,000 skeleton data points and 230,000 acceleration values. The system setup includes two inertial measurement units (IMUs) mounted on the subject’s wrist and waist, along with a Microsoft Kinect v2 sensor. Additionally, the fall detection system utilizes IMU sensor data and depth information obtained by the Microsoft Kinect v2 sensors. A shimmer device is positioned on the right side of the body, secured at the waist with a belt, while another accelerometer simulates a smartwatch on the right wrist. The Kinect sensor monitors the test area from a distance of approximately 1.5 m above the floor and 2 m away from the person. Notably, the dataset also includes 1,594 RGB-D images extracted from this meticulous data collection effort and used in the proposed fall detection system.

### 3.3 Experimental results

In the following section, we evaluate the benchmark datasets using the receiver operating characteristic (ROC) curve, error bar graph, and leave-one-out cross-validation (LOSO) scheme. Additionally, we validate the proposed model on real-world data by collecting IMU sensor readings from the chest, elbow, and ankle positions of two individuals, referred to as person 1 and person 2. Further details are provided in the subsequent sections.

#### 3.3.1 Error bar and ROC results on benchmark datasets

**FIGURE 12:**
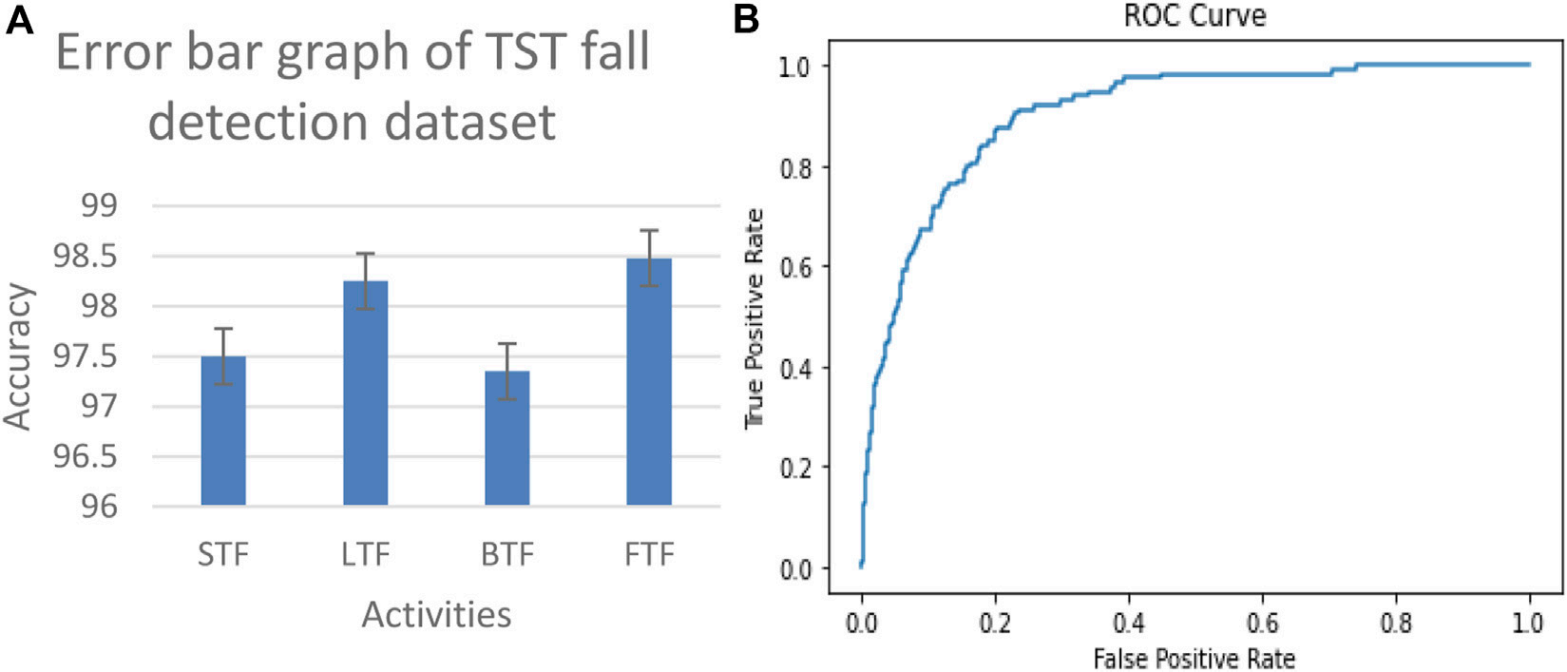
Error bar graph and receiver operating characteristic (ROC) curve results of the TST Fall Detection dataset.

**FIGURE 13:**
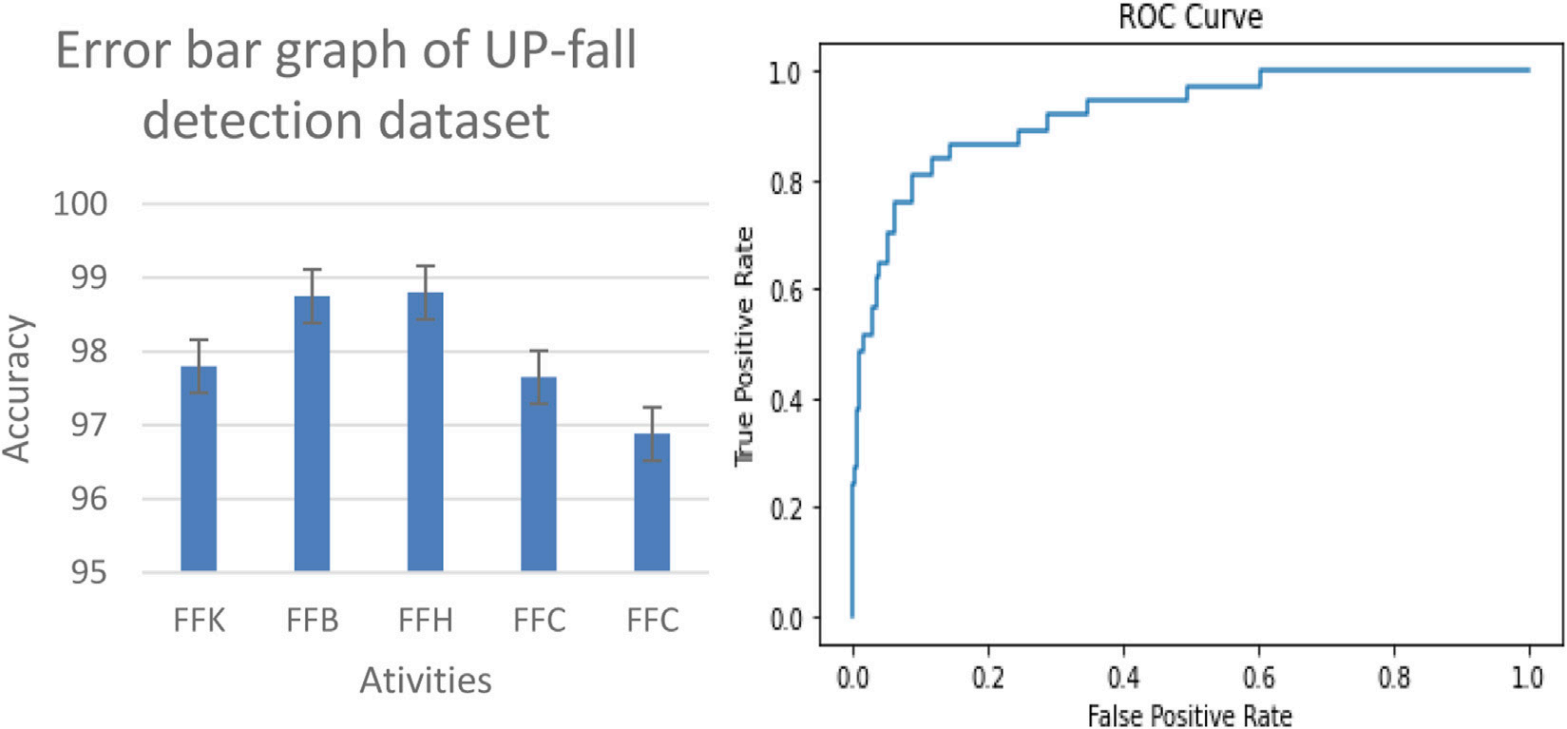
Error bar graph and ROC curve results of the UP-Fall Detection dataset.

The proposed system is evaluated by using the ROC curve and error bar graph. The ROC curve illustrates the diagnostic ability of our proposed system as its discrimination threshold is varied. Each point on the ROC curve represents a sensitivity/specificity pair corresponding to a particular decision threshold. The area under the ROC curve (AUC) provides a single scalar value to summarize the overall performance of the model. A higher AUC indicates better performance, with a value of 1 representing a perfect model. In addition to analyzing the ROC curve, we used an error bar graph to assess the performance of our fall detection system. This graph visually represents the prediction variability and reliability of the model. Each bar corresponds to the mean accuracy for detecting falls and non-falls, while the error bars indicate the standard deviation or confidence intervals around these means. Essentially, it shows the range within which the true accuracy likely lies, reflecting the model consistency across various trials or data samples. The results of the TST Fall Detection dataset and UP-Fall Detection dataset are shown in Figures 12, 13, respectively.

#### 3.3.2 Error bar and ROC results on real-world data

**FIGURE 14:**
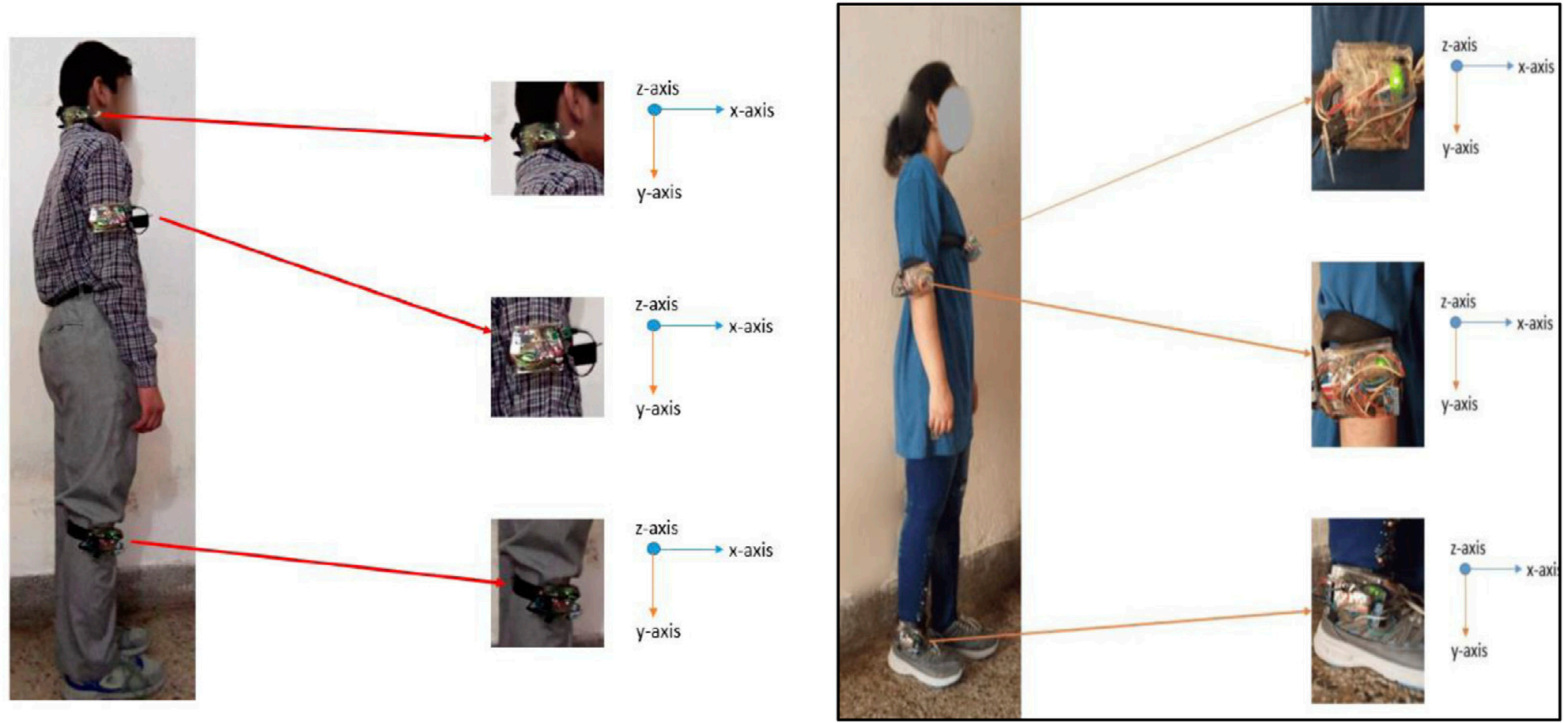
Wearable sensors positioned on person 1 and person 2 (from left to right) at the chest, elbow, and ankle.

**FIGURE 15:**
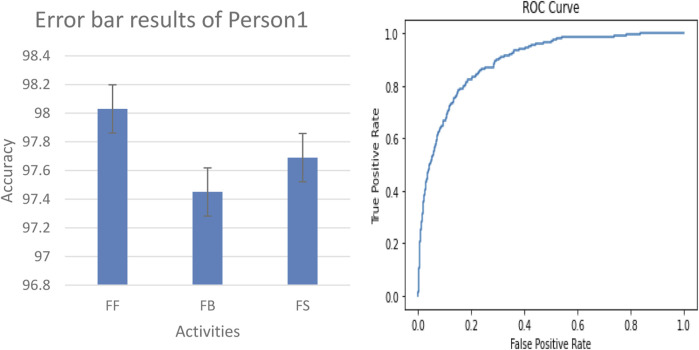
Error bar graph and ROC curve results of person 1.

**FIGURE 16:**
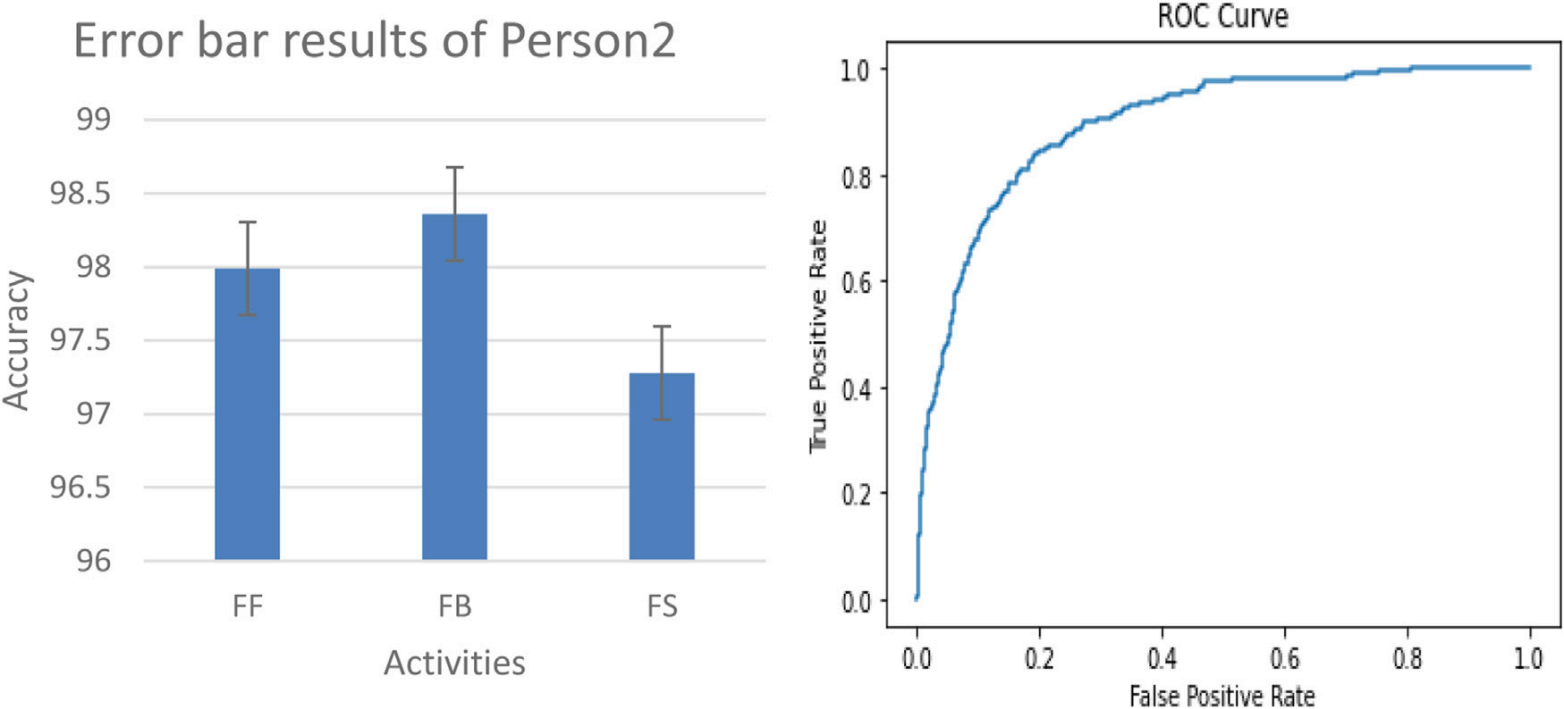
Error bar graph and ROC curve results of person 2.

In our evaluation of the fall detection system using real-world data, we collected IMU sensor readings located at the chest, elbow, and ankle positions of person 1 and person 2, as shown in Figure 14. The physical activities include falling forward (FF), falling backward (FB), and falling sideway (FS). We generated a ROC curve and error bar graph, as shown in Figures 15, 16, respectively. Each point on the ROC curve corresponds to a sensitivity/specificity pair. Our model achieved a mean accuracy of 97% across the three real-world activities, effectively distinguishing between fall and non-fall events. The results of the ROC curve and error bar graph demonstrate the ability of the model to identify true positives while minimizing false positives. The results indicate that the robustness of our model is crucial for real-world applications, improving the response time, model stability, precision, and outcomes for at-risk individuals.

#### 3.3.3 LOSO results on benchmark datasets

The performance of the proposed model has been assessed using the LOSO scheme to evaluate the average accuracy of benchmark datasets. The average accuracy of UP-Fall Detection and TST Fall Detection datasets is given in Tables 1, 2, respectively. Moreover, the results of precision, F1 score, and recall (Rafique et al., 2019) are given in Tables 3, 4 respectively. Finally, the comparison of the proposed methodology with other cutting-edge methods over UP-Fall Detection and TST Fall Detection datasets is given in Tables 5, 6, respectively.

**TABLE 1:** Confusion matrix results of the UP-Fall Detection dataset.

UP-Fall Detection dataset	FFK	FFB	FFH	FFC	FFS
FFK	97.80	0.5	0.2	0.5	1.0
FFB	0	98.75	0.5	0.25	0.5
FFH	0.2	0	98.80	0.5	0.5
FFC	0.5	1.5	0.15	97.65	0.2
FFS	0.1	0.02	1.5	1.5	96.88
Average recognition accuracy = 97.97%					

FFK, falling forward on knees; FFB, falling backward; FFH, falling forward on hands; FFC, falling while sitting on an empty chair; FFS, falling sideward.

**TABLE 2:** Confusion matrix results of the TST Fall Detection dataset.

TST Fall Detection dataset	STF	LTF	BTF	FTF
STF	97.50	0.5	2.5	0.5
LTF	0	98.25	1.7	0.05
BTF	2.0	0.6	97.35	0.05
FTF	0.5	1.02	0	98.48
Average recognition accuracy = 97.97%				

STF, sitting fall; LTF, lateral fall; BTF, backward fall; FTF, front fall.

**TABLE 3:** Precision, recall, and F-measure results obtained over the UP-Fall Detection dataset using the LOSO scheme.

UP-Fall Detection dataset	Precision	Recall	F-measure
FFK	0.978	0.974	0.975
FFB	0.987	0.980	0.983
FFH	0.988	0.987	0.987
FFC	0.976	0.978	0.976
FFS	0.968	0.969	0.968
Average	0.979	0.977	0.977

**TABLE 4:** Precision, recall, and F-measure results obtained over the TST Fall Detection dataset using the LOSO scheme.

TST Fall Detection dataset	Precision	Recall	F-measure
STF	0.975	0.980	0.977
LTF	0.982	0.985	0.983
BTF	0.973	0.970	0.971
FTF	0.984	0.981	0.982
Average	0.978	0.979	0.978

**TABLE 5:** Comparison of the recognition accuracy of the proposed model with cutting-edge techniques over the UP-Fall Detection dataset.

Author	Method	Accuracy (%)
Ghadi et al. (2022)	Hidden Markov model (HMM)	87.50
Villaseñor et al. (2019)	Random forest (RF)	95.10
Marvi et al. (2021)	Bidirectional long short-term memory	97.41
Kraft et al. (2020)	Deep learning algorithm	93.30
Proposed approach	Modified K-Ary entropy (KEC)	97.97

**TABLE 6:** Comparison of the recognition accuracy of the proposed model with cutting-edge techniques over the TST Fall Detection dataset.

Author	Method	Accuracy (%)
Gasparrini et al. (2016)	Data fusion	90.00
Yao et al. (2019)	Support vector machine (SVM)	93.56
Seredin et al. (2019)	Encoding features	95.80
Mendez et al. (2019)	Genetic + principal component analysis (PCA) + SVM	90.00
Proposed approach	Modified K-Ary entropy (KEC)	97.89

## 4 Conclusion

In this paper, we introduced a comprehensive multimodal approach for fall detection. Our method integrates data from RGB, depth, and inertial sensors to enhance accuracy and reliability. The pre-processing stage effectively removes noise from each sensor type, ensuring clean input data. For feature extraction, we used autoregressive and waveform length features for inertial data while using MRF and ridge methods for depth and RGB data, respectively. To fuse the results obtained from multimodal sensors, we utilized PCA, which facilitated robust classification. For the final classification, the modified K-Ary entropy classifier was used, achieving superior performance. Evaluation using LOSO on the UP-Fall Detection and TST Fall Detection datasets demonstrated the efficacy of our approach. Our method surpassed the state-of-the-art methods with impressive accuracy, precision, recall, and F-measure scores of 97.97% and 97.89%. These results underscore the effectiveness of our proposed multimodal approach in fall detection applications. The proposed fall detection model will help in enhancing the safety of elderly people by generating an alert to caregivers during a fall, ensuring quick assistance. Moreover, it will promote independence, reduce the need for constant caregiver monitoring, help prevent costly hospitalizations, and can also be integrated with smart home devices through AI advancements.

In the future, we aim to develop our own comprehensive elderly fall dataset, encompassing diverse scenarios and environmental conditions relevant to fall detection. This dataset will incorporate a variety of sensors to capture a wide range of fall-related events and contexts accurately. Additionally, we intend to validate the effectiveness and robustness of our proposed technique across various benchmark fall detection datasets. This validation will help further establish the generalization and reliability of our approach across different settings and populations.

## Data Availability

Publicly available datasets were analyzed in this study. These data can be found at: https://github.com/hoangNguyen210/Fall-Detection-Research-1, https://sites.google.com/up.edu.mx/har-up/, https://ieee-dataport.org/documents/tst-fall-detection-dataset-v2.
